# Prognostic intraoperative factors in severe acute pancreatitis

**Published:** 2014

**Authors:** CC Popa

**Affiliations:** * Carol Davila” University of Medicine and Pharmacy, Bucharest, Romania; Department of Surgery, 2nd Surgery Clinic, University Emergency Hospital Bucharest, Romania

**Keywords:** acute pancreatitis, severity, prognosis, intraoperative

## Abstract

Acute pancreatitis is a serious disease. Triggered by the local inflammation of the pancreas, it can cause inflammation in various organs and systems in the body. It is important to identify severe forms of acute pancreatitis with an increased morbidity and mortality rate. Lately, internationally, numerous clinical and paraclinical factors predicting the severity of acute pancreatitis have been proposed. The purpose of the study is to identify the prognostic intraoperative factors of severity. The prospective study was conducted over a period of four years, between 2007 and 2010 and included 238 patients treated in a surgical clinic in Bucharest. 103 patients experienced a severe form of acute pancreatitis, which means 67.95% of all operations practiced. We monitored intraoperative factors, in particular: the presence and/ or the extent of pancreatic necrosis, common bile duct lithiasis and intraperitoneal fluid, parameters proposed to become statistically prognostic factors in the development and long-term morbidity of acute pancreatitis. The presence and/ or extension of necrosis was identified in the histopathology only in patients with severe acute pancreatitis. 71.43% of the patients with common bile duct lithiasis and 73.91% of the patients with inflammatory intraperitoneal fluid had severe acute pancreatitis. Most patients who developed postoperative complications (86.49%) or who required a surgical intervention (85.71%), presented a severe form of the disease.

Conclusions: pancreatic necrosis, common bile duct lithiasis and intraperitoneal fluid may contribute to a more precise prediction of severity, as confirmed by international literature.

## Introduction

Acute pancreatitis is the acute inflammation of the pancreas gland acinar cells, with consequent autodigestion of the local tissue destruction followed by ischemic necrosis. If pathology worsens, the inflammatory reaction becomes massive, pancreatic enzymes are released into the circulation and peripancreatic tissues and other organs and systems in the body will be affected. It will trigger the systemic inflammatory response syndrome, which causes multiple organ dysfunctions, which are the leading cause of morbidity and mortality in acute pancreatitis. To estimate the severity of acute pancreatitis, it is important to differentiate the mild forms of the severe evolving that have a serious trend and increased mortality. In recent years, multiple prognostic factors in severe acute pancreatitis have been found. Our prospective study was done over a period of four years, between 2007 and 2010. The objective was to present the experience of a general surgery clinic of an emergency hospital in Bucharest, regarding the identification of the intraoperative prognostic factors in severe acute pancreatitis.

The positive diagnosis and assessment of severity of acute pancreatitis was made on etiological, clinical, biological, imaging, intraoperative and pathological criteria. In the present study, numerous intraoperative data, especially pancreatic necrosis, intraperitoneal inflammatory fluid and common bile duct lithiasis were followed.

The statistical analysis was performed by including monitoring data, to observe the role of the intraoperative risk factors on the development of mild or severe acute pancreatitis. We used SPSS 10.0 statistical software for Windows to coordinate data and statistical tests, with Word, Excel and Epi Info software. Data were expressed as numbers, averages and percentages. 

238 patients diagnosed with acute pancreatitis and its complications were studied and treated in the clinic during 2007-2010. Of all the patients, 135 (56.72%) had mild acute pancreatitis and 103 patients (43.28%) had severe acute pancreatitis.

In terms of gender distribution of the 103 patients with severe acute pancreatitis, 58 were men (56.31%) and 45 women (43.69%). Pathology seemed to affect in some way more than men.

The average age of patients in the study group was 52.43 years and had limits between 23 and 84 years. Distributed according to gender, the average age of women was 55.36 years, slightly higher than that of men, of 50.38 years. Depending on the severity of acute pancreatitis, the median age in patients with mild was 50.5 years, slightly lower than in patients with the severe form, which was 55.0 years.

78 surgical interventions were performed and intraoperative obtained data were analyzed. Of the total operations, 25 were performed for mild acute pancreatitis (32,05%) and 53 for severe acute pancreatitis (67.95%). Beger procedure was the most commonly used and consisted of: pancreatic necrosectomy, lesser sac and possible necrotic cavity drainage and closing or leaving open the abdomen.

An important intraoperative note which was observed was the impaired pancreas. Of the 78 patients operated on, in 45 (57.69%), an enlarged pancreas was found. Depending on the topography, a cephalic impaired pancreas was found in 11 patients (24.44%), the body in 5 patients (11.11%), the tail in 3 patients (6.67%), and the entire pancreas in 26 patients (57.78%). Other pancreatic morphological changes observed intraoperatively were pancreatic tenderness in 15 patients (19.23%), pancreatic edema in 22 patients (28.21%), edematous-hemorrhagic appearance in 8 patients (10.26%), pancreatic necrosis in 22 patients (28.21%), necrotic hemorrhagic appearance in 25 patients (32.05%) and hemorrhagic appearance itself in one patient (1.28%).

**Fig. 1:**
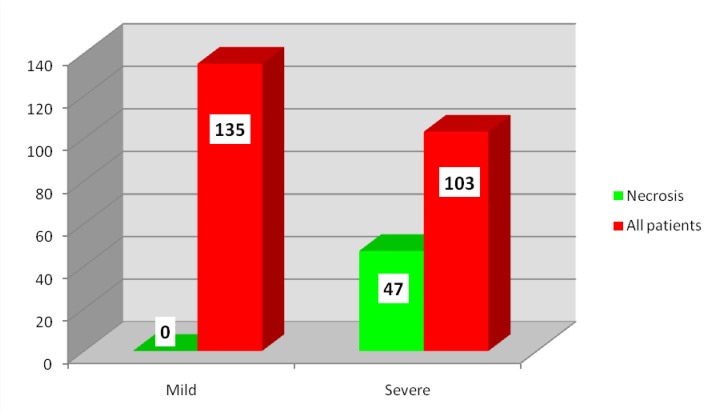
Distribution of patients according to the presence and/ or extension of necrosis and severity

The presence and/ or extension of necrosis was identified in histopathology only in patients with severe acute pancreatitis. Of the 103 patients with severe acute pancreatitis, in 47 patients (45.63%), pancreatic necrosis was identified afterwards as shown in **Fig. 1**.

6 patients were found with a limited pancreatic necrosis (with diffuse bleeding or marked edema). The extension of the retroperitoneal necrosis was observed in 6 patients: based on the mesentery (3 patients), colo-parietal space, exactly right retrocolic (1 patient) and the transverse mesocolic (2 patients). Cytosteatonecrosis stains were present in the peritoneum (12 patients), the great omentum (3 patients) and the transverse mesocolic (2 patients).

Pancreatic collections were found in 27 of the operated patients (34.62%). Of these, 13 patients had pseudocysts (48.15%), distributed according to the location in the pancreas as it follows: 6 patients in the tail (46.15%), 4 patients in the body (30.77%) and 3 in the head (23.08%).

Of the 78 patients operated on, peripancreatic collections (32.05%) were found intraoperatively in 25. Of these, the dissemination of peripancreatic collections was found in 17 patients (68.0%).

**Fig. 2:**
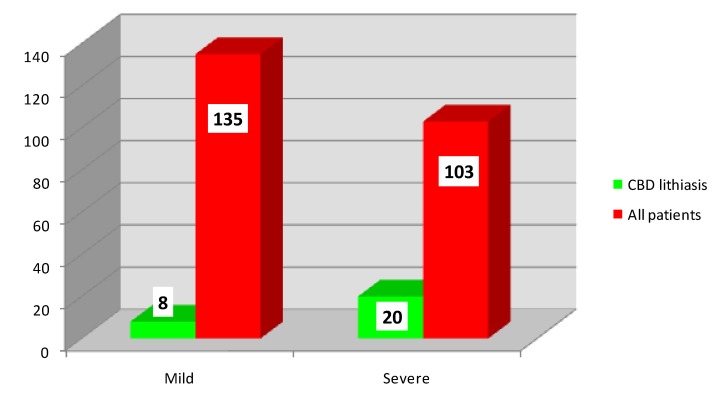
Distribution of patients according to common bile duct lithiasis and severity

While performing biliary surgery, gallbladder lithiasis was found in 38 patients who underwent cholecystectomy (48.72%). Common bile duct lithiasis was present in 28 patients (35.89%). Of these, 8 patients had mild acute pancreatitis (28.57%) and 20 patients severe (71.43%), as shown in **Fig. 2**.

In 23 patients, biliary drainage (29.49%) was established.

**Fig. 3:**
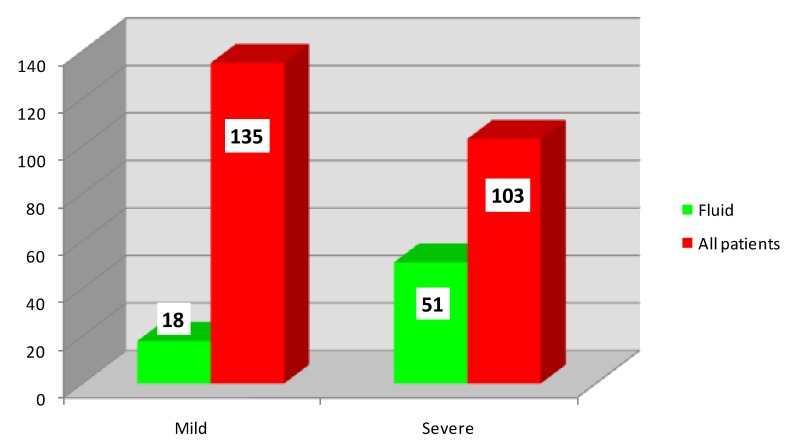
Distribution of patients according to the intraperitoneal inflammatory fluid and severity

The intraperitoneal inflammatory fluid was observed in 69 patients (88.46%), 18 patients with mild (26.09%) and 51 patients with severe acute pancreatitis (73.91%). Compared to the severity of acute pancreatitis, in 103 patients with a severe form, 51 patients (49.51%) showed fluid, and from 135 patients with a mild form, only 18 patients (13.33%) showed fluid (**Fig. 3**).

Intraoperative intraperitoneal fluid was found in many types: hemorrhagic (in 23 patients), hemorrhagic-dark (in 2 patients), hemorrhagic-gray (in 2 patients), brown (in 7 patients), pus-cloudy (in 19 patients), hemorrhagic-cloudy (in 2 patients), ascites (in 3 patients), serohematic, slightly turbid (in 4 patients), bilious (in 2 patients), serous, clear (in 5 patients). The graph below (**Table 1**) shows mainly hemorrhagic fluid or pus identified intraoperatively.

**Table 1:** The correlation between the type and amount of intraperitoneal fluid

	Hemorrhagic	Pus	Serous	Bilious
Small	9	7	4	2
Medium	10	13	5	0
Large	8	8	3	0

The pancreatic lodge drainage was performed in 38 patients (48.72%) and the peritoneal cavity drainage in 74 patients (94.87%). Peritoneal drainage was multiple in 62 patients (83.78%) and unique in 12 patients (16.22%).

The radiographic evaluation revealed the pleural fluid. In 15 patients, the presence of pleural fluid was revealed, and a patient’s fluid was present bilaterally. Of the 16 patients who had pleural reaction, 4 patients received left pleural drainage and one patient a bilateral pleural drainage. Of the four pleurostomia for the left side, three of them evacuated serous-citrine fluid and one yellow-brown fluid. The bilateral pleural drainage evacuated serous-citrine fluid, the amount of 1600 ml on the left side and 800 ml on the right side.

**Fig. 4:**
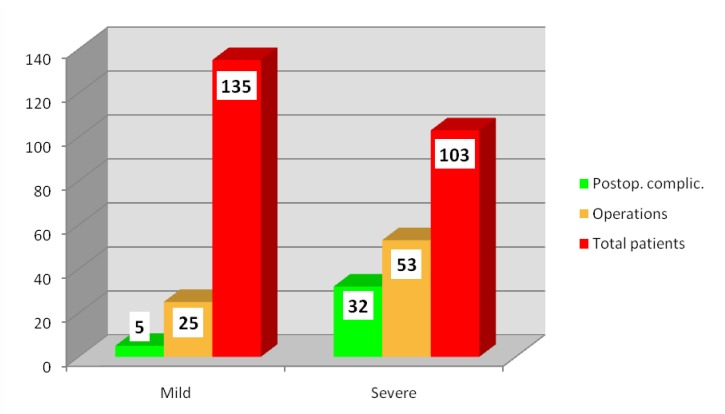
Distribution of patients according to postoperative complications, number of operations and severity

Compared to the 87 operations, postoperative complications were present in 37 patients (47.44%), 32 patients with severe acute pancreatitis (86.49%) and only 5 patients with mild acute pancreatitis (13.51%), as shown in **Fig. 4**.

Postoperative complications were due to certain factors: the surgical technique used, if any; the extension of necrosis; an incomplete treatment performed.

The surgical reintervention was required in 14 patients (17.95%), 12 patients with severe (85.71%) and only 2 patients with mild (14.29%) acute pancreatitis (**Table 2**).

**Table 2:** Number of reinterventions performed according to the clinical form of acute pancreatitis

	Mild acute pancreatitis	Severe acute pancreatitis	Total patients
Number of patients	135 (56,72%)	103 (103/238; 43,28%)	238 (238/238; 100,00%)
Actual operations	22 (22/64; 34,38%)	42 (42/64; 65,63%)	64 (64/78; 82,05%)
Reinterventions	2 (2/14; 14,29%)	12 (12/14; 85,71%)	14 (14/78; 17,95%)
Total operations	25 (25/78; 32,05%)	53 (53/78; 67,95%)	78 (78/238; 32,77%)

Of the 14 patients who required reoperation, only 6 patients (7.48%) survived the intervention, the other 8 patients (10.47%) died, pathology examination confirming extensive necrosis.

## Discussion. Conclusion

• The average age of the patients in the study group was 52.43 years, similar to the international literature [**1**].

• Most surgical procedures (66%) were performed in patients with severe acute pancreatitis, according to the international guidelines for treatment recommendations [**2**].

• The latest international classification of acute pancreatitis, revision of the Atlanta classification, expressed by Acute pancreatitis Classification Working Group on international consensus in 2012, showed that both forms of acute pancreatitis moderately severe and severe include local complications (necrosis, collections). For this reason, it is important to identify local complications because they require a therapeutic range to avoid the fatal evolution [**3**].

• An important prognostic factor for the severe disease is the presence and/ or necrosis extending on a variable period of time. The existence of pancreatic necrosis in patients with mild acute pancreatitis was not proved, which demonstrated the importance of the presence/ extension of necrosis in severe morbidity and mortality of the disease. Although the presence of intraoperative pancreatic necrosis is not identified, the preoperative imaging confirms the severity of the disease. A negative fine needle aspiration with imaging guidance does not exclude infection. The clinical status of the patient is more important in deciding the surgical debridement of the infected necrosis and not necessarily the proof of infection. The infected pancreatic necrosis was found intraoperatively in a higher proportion than preoperatively [**4**,**5**].

• Studies have shown that the open transperitoneal debridement followed by draining and abdominal wall closure is a standard, as compared to the other methods of treatment [**4**]. Analyzing the Romanian literature, Popa published a study in 2013 about the importance of debridement and notes regarding the avoidance of septic remnants which presuppose the insistence on six access pathways to the pararenal space [**6**]. On the other hand, Constantinoiu published a study in 2014 and proposed a partially open surgical drainage, using the open packing of the lesser sac technique in severe acute infected pancreatitis [**7**].

• It is known in the international literature that persistent gallstones in the common bile duct are associated with severe episodes of acute pancreatitis [**8**,**9**], as confirmed in our study.

• The intraoperative presence of intraperitoneal inflammatory fluid is closely associated with the severe evolution of the disease, as confirmed by various international clinical and paraclinical scores of assessing the severity of acute pancreatitis [**10**,**11**]. Furthermore, Costea and Neagu affirm that the abdominal compartment syndrome caused by the accumulation of intraperitoneal fluid along with other factors, is an independent factor increasing mortality [**12**]. It is remarkable that intraperitoneal fluid can be infected in the presence of sterile necrosis [**13**].

• Postoperative complications, mainly due to multiple organs dysfunction syndrome, were associated with a severe disease, as confirmed by international studies [**4**].

• In conclusion, our analysis confirms the existence of various intraoperative factors for the most accurate prediction of severity, as confirmed by different authors in Romanian or international literature.
